# Comparative survival benefit of currently licensed second or third line treatments for epidermal growth factor receptor (EGFR) and anaplastic lymphoma kinase (ALK) negative advanced or metastatic non-small cell lung cancer: a systematic review and secondary analysis of trials

**DOI:** 10.1186/s12885-019-5507-6

**Published:** 2019-04-25

**Authors:** Martin Connock, Xavier Armoiry, Alexander Tsertsvadze, G. J. Melendez-Torres, Pamela Royle, Lazaros Andronis, Aileen Clarke

**Affiliations:** 1 Warwick Medical School, Division of Health Sciences, University of Warwick, Gibbet Hill road, CV47AL Coventry, England; 2 School of Pharmacy (ISPB) / UMR CNRS 5510 MATEIS / Lyon University Hospitals, Edouard Herriot hospital, Pharmacy Department, University of Lyon, 8 avenue Rockefeller, 69008 Lyon, France; 3 School of Epidemiology and Public Health, University of Ottawa, Ottawa, Canada; 4 Peninsula Technology AssessmentGroup (PenTAG), University of Exeter, Exeter, UK

**Keywords:** Advanced NSCLC, Licensed drugs, Targeted therapies, Immunotherapies, Check point inhibitors

## Abstract

**Background:**

A review of therapies for advanced cancers licenced by the EMA between 2009 and 2013 concluded that for more than half of these drugs there was little evidence of overall survival or quality of life benefit. Recent years have witnessed a growing number of licensed second-line pharmacotherapies for advanced/metastatic non-small cell lung cancer (NSCLC). With the aim of gauging patient survival benefit, we conducted a systematic review of randomised controlled trials (RCT) and compared survival outcomes from available licensed treatments for patients with advanced/metastatic NSCLC.

**Methods:**

RCTs of second/third line treatments in participants with advanced/metastatic NSCLC and negative/low expression of Anaplastic Lymphoma Kinase (ALK) and of Epidermal Growth Factor Receptor (EGFR) were included. We searched electronic databases (MEDLINE; EMBASE; Web of Science) from January, 2000 up to July, 2017. Two or more independent reviewers screened bibliographic records, extracted data, and assessed risk of bias of studies. Published Kaplan Meier plots for OS and PFS along with restricted-mean-survival methods and parametric modelling were used to estimate the survival outcomes as mean number of months of survival. Network meta-analysis was undertaken to rank interventions and to make indirect comparisons.

**Results:**

We included 11 RCTs with data for 7581 participants that compared nine different drugs. In studies of patients regardless of histology groups, targeted drugs (ramucirumab and nintedanib) yielded small overall survival gains of < 2.5 months over docetaxel, erlotinib provided no benefit, while immunotherapies (atezolizumab and pembrolizumab) delivered 5 to 6 months gain. Studies with patients stratified by histology confirmed the apparent superiority of immunotherapy (nivolumab and atezolizumab) over targeted treatments (ramucirumab, nintedanib, afatinib) providing between about 4 to 8 months OS gain over docetaxel. In network analysis immunotherapies consistently ranked higher than alternatives irrespective of population histology and outcome measure.

**Conclusion:**

Our review indicates that nivolumab, pembrolizumab and atezolizumab provide superior survival benefits compared to other licensed drugs for late stage NSCLC. Patient gains from these immunotherapies are substantial compared to the expected average survival with chemotherapy (docetaxel) of < 1 year for people with squamous histology and about 1.25 year for those with non-squamous histology.

**Electronic supplementary material:**

The online version of this article (10.1186/s12885-019-5507-6) contains supplementary material, which is available to authorized users.

## Background

Lung cancer is the second most common cancer in both men and women. [1] It is the leading cause of cancer death in both men and women. Excluding mesothelioma, non-small cell lung cancer (NSCLC) accounts for about 85% of all lung cancers. [2] Many patients have a delayed diagnosis and are unsuitable for surgery so that most receive some form of first line pharmacotherapy. In the past, following failure of first-line therapies most NSCLC patients received docetaxel [3], however in recent years targeted therapies and immunotherapies have been developed, the latter acting as immune checkpoint inhibitors with the aim of boosting anti-tumour immunity rather than directly targeting cancer cells. About 12 agents now have a label indication for second- or further line NSCLC treatment. A 2017 study [4] of cancer drugs approved by the EMA from 2009 to 2013 concluded that most of these drugs entered the market without evidence of benefit on survival or quality of life, and that after a median of 3.3 years post-market entry, there was little or no conclusive evidence of extended or better life for most cancer indications.

The effectiveness of the second line new agents for treating NSCLC in absolute terms is unknown because previous trial analyses focused mostly on the relative benefit (versus standard chemotherapy mainly consisting of docetaxel), usually expressed in terms of OS and PFS hazard ratios [5, 6].

In this systematic review we estimated the survival benefit (i.e. mean number of months) from licensed therapies for NSCLC. It is hoped that the findings focused on new drugs may contribute to more informed discussion between patients and clinicians and will support the decision-making process.

## Methods

We registered a protocol for this review in PROSPERO (CRD42017065928).

### Inclusion/exclusion criteria

We included RCTs of adult patients with advanced or metastatic (IIIB and/or IV) NSCLC with non-squamous (adenocarcinoma, large cell) or squamous histology who had experienced failure to prior first line chemotherapy (i.e., those receiving second line treatment and beyond); had either predominantly negative or 100% negative expression of anaplastic lymphoma kinase (ALK); had either predominantly negative or 100% negative expression of epidermal growth factor receptor (EGFR). Studies enrolling only patients with ALK+ and/or EGFR+ expression were excluded since according to current practices they would be offered targeted therapies (erlotinib or gefitinib for EGFR+; osimertinib for EGFR T790 M; crizotinib or ceretinib for ALK+). [1]

RCTs were included if interventions or comparators had an EMA (European Medicines Agency) label indication as of June, 2017 for the population described above. The drugs meeting these criteria were Docetaxel (DOC), Pemetrexed (PEM), Ramucirumab plus docetaxel (RAM + DOC), Erlotinib (ERL), Nintedanib plus docetaxel (NIN + DOC), Afatinib (AFA), Nivolumab (NIVO), and Pembrolizumab (PEMBRO). We also included Atezolizumab (ATEZO) which obtained an EMA license following the Committee for Medicinal Products for Human Use (CHMP) positive opinion of 20 July 2017. Only studies in which drugs were used with a dose regimen as described in the summary of product characteristics were included. The following drugs such as Crizotinib, Ceretinib, Gefetinib, Osimertinib which are used in people with ALK+ and/or EGFR+ expression were excluded.

Studies were included if either an overall survival or progression-free survival or both parameters were reported in published Kaplan-Meier plots.

### Search strategy

Electronic databases (MEDLINE; EMBASE; Web of Science) were searched for relevant literature from January, 2000 up to present (see MEDLINE search strategy in Additional file 1). The electronic searches were limited to English language. The lower time limit for the search period was chosen in accordance with the emergence of docetaxel as the standard second-line treatment. Reference lists of relevant articles were hand-searched to identify additional potentially relevant citations. The search was first updated up to early July 2017 retrieving 274 additional records but no further studies were included. A final update of the search was undertaken up to February 2019 to identify additional original articles relevant to the included studies. The latter retrieved 651 records of which six were selected for further scrutinity.

### Selection of studies

Three reviewers independently screened all titles/abstracts and then full texts of publications potentially relevant for inclusion. Disagreements were resolved through a consensus. The study flow and reasons for exclusion at the full text screening level are presented in the PRISMA study flow diagram [7] (Additional file 2).

#### Data extraction

The data extracted included study author, trial acronym, patient characteristics (age, sex, diagnosis, tumour stage/histology), type, mode, dose and duration of treatments. Extracted data was cross-checked by a second reviewer.

Published Kaplan-Meier (KM) survival plots were used to make estimates of mean survival benefit. Two reviewers digitised the KM plots, extracted patient numbers at risk, numbers of events, and published hazard ratios.

#### Assessment of risk of bias

Two independent reviewers assessed the risk of bias (RoB) in the included studies using the Cochrane RoB tool for RCTs; [8] this categorises studies according to the following domains of potential bias: selection bias (random sequence generation, allocation concealment), performance bias (blinding participants and personnel), detection bias (blinding of outcome assessment), attrition bias (incomplete outcome data), reporting bias (selective outcome reporting), and “other” bias (e.g. between-group baseline distribution of important prognostic factors). Summary ratings of high RoB were assigned if at least one of the domains of selection, attrition, and other bias was rated as high RoB. If information was insufficient to judge, then an unclear RoB rating was assigned. Quality assessment was performed by two independent reviewers and then cross-checked. Any disagreements were resolved by a third reviewer through a discussion.

##### Data analysis and synthesis

We used the algorithm of Guyot et al. [9] to estimate underlying individual patient data, which was then used to reconstruct KM plots and to derive estimates of mean survival. The reliability of KM reconstructions was tested by inspection of reconstructions overlaid onto published plots, comparison of reconstructed and published risk table of patients at risk, and correspondence of reconstructed HRs with published HRs (Additional file 3).

Mean survival was estimated in several ways. Restricted mean survival (RMS) [10] and mean difference in RMS between compared drugs in each trial, were estimated to the longest time common across the compared studies of interest using the Stata module of Cronin et al. 2016 [11].

In order to account for any potential gains beyond the longest observation time common across trials, we undertook analysis of total mean survival using parametric survival modelling. Total mean survival was estimated: [a] with Weibull models (fit separately by study arm) using the *stgenreg* package of Crowther and Lambert 2013 [12]; mean survival time and 95% confidence intervals (CIs) were estimated from the AUC of the model and its upper and lower 95% CIs using 0.01 month increments over 96 months. The CIs around the central AUC estimate were somewhat asymmetric (as would be expected from the delta method for estimating CIs around parametric models). The SE for the AUC value was therefore estimated from the difference between 95% LCI and UCI AUC values divided by 2 × 1.96. In two instances Weibull models were inferior to generalised gamma models in which case the latter were used; [b] Total mean survival was also calculated using the equations for mean survival published by Davies et al. 2012 [13] for Weibull parametric survival models; [c] Lastly, total mean survival was also estimated using the “*stci, emean”* command in Stata; this command uses an exponential extension from the tail of the KM plot to the time axis; and mean survival is then estimated from the AU the KM plot plus that under the extension. Similar methods were applied for progression free survival (PFS) (Additional file 4).

We did exploratory analyses to investigate the relationships between PFS and RMS and modelled total survival, and between published hazard ratios and median survival values and RMS and modelled total survival.

Analyses were done using Stata® versions 12 or 14.2 (Stata Corp, College Station, TX, USA).

The outcome estimates are presented in KM plots, model plots, forest plots, and tables. Where possible, the analyses were stratified by histologic subtypes (squamous and non-squamous).

For completeness, we undertook a network meta-analysis to estimate the mean differences in RMS and in OS. The description of corresponding methods was reported as Additional file 7.

## Results

Our search retrieved 1949 records, of which 1855 were excluded at title/abstract level leaving 94 records to be examined for full-text. We subsequently excluded 81 records with reasons as illustrated on the PRISMA flow chart and included 13 records [14–26], corresponding to 11 primary RCT studies with 7581 participants (REVEL, LUME LUNG-1, LUX LUNG 8, OAK, POPLAR, KEYNOTE-01, CHECKMATE-017, CHECKMATE-057, TAILOR, HORG, and Hanna et al. 2004). No studies were omitted because of a lack of KM plot, but some included studies did not provide plots for all histology subgroups.

### Study characteristics and quality

The 11 RCTs compared nine different drugs with the majority of comparisons were against DOC. Two comparisons, ATEZO vs DOC [17, 24] and NIVO vs DOC [14–16] were tested in more than one study. The NIVO studies employed histology-specific inclusion criteria. Table 1 summarises the main characteristics reported for the 11 studies. Study sample size ranged from 208 to 1314 patients; studies included predominantly people with stage IV NSCLC and performance status 1. The mean age at inclusion ranged from 57 to 66 years and the majority of patients were male. There was no evidence of substantial imbalance in potential effect modifiers.

**Table 1:** Characteristics of included studies

Variables *n* % unless stated	REVEL		LUME-LUNG 1		CHECKMATE 017		CHECKMATE 057		Hanna		KEYNOTE-010		POPLAR		TAILOR		OAK		Lux-Lung 8		HORG	
	RAM + DOC *n* = 628	PBO + DOC *n* = 625	NIN + DOC *n* = 655	PBO + DOC *n* = 659	NIVO *n* = 135	DOC *n* = 137	NIVO *n* = 292	DOC *n* = 290	PEME *n* = 283	DOC *n* = 288	PEMB *n* = 344	DOC *n* = 343	ATEZ *n* = 144	DOC *n* = 143	ERLO *n* = 109	DOC *n* = 110	ATEZ *n* = 425	DOC *n* = 425	AFA *n* = 398	ERL *n* = 397	PEME *n* = 166	ERL *n* = 166
Age, years median, *range*	62 ***21–85***	61 ***25–86***	60 ***53–67***	60 ***54–66***	62 ***39–85***	64 ***42–84***	61 ***37–84***	64 ***21–85***	59 ***22–81***	57 ***28–87***	63 ***56–69***	62 ***56–69***	62 ***42–82***	62 ***36–84***	66 ***40–81***	67 ***35–83***	63 ***33–82***	64 ***34–85***	65 ***36–84***	64 ***35–88***	66 ***42 86***	65 ***37–83***
Male sex	419 ***67***	415 ***66***	476 ***73***	479 ***73***	111 ***82***	97 ***71***	151 ***52***	168 ***58***	194 ***68.6***	217 ***75.3***	212 ***62***	209 ***61***	93 ***65***	76 ***53***	77 ***71***	73 ***66***	261 ***61***	259 ***61***	335 ***84***	331 ***83***	138 ***83.1***	135 ***81.3***
White	526 ***84***	503 ***81***	533 ***81***	530 ***80***	122 ***90***	130 ***95***	267 ***91***	266 ***92***	NA	NA	246 ***72***	251 ***73***	NR	NR	108 ***99***	109 ***99***	302 *71*	296 ***70***	312 ***78***	311 ***78***	NR	NR
Asian	74 ***12***	86 ***14***	116 ***18***	123 ***19***	4 ***3***	2 ***1***	9 ***3***	8 ***3***			73 ***21***	72 ***21***	NR	NR	1 ***1***	1 ***1***	85 *20*	95 ***22***	86 ***22***	86 ***22***	NR	NR
Black	17 ***3***	16 ***3***	4 ***< 1***	5 ***< 1***	6 ***4***	2 ***1***	7 ***2***	9 ***3***			13 ***4***	7 *2*	NR	NR	0	0	5 *1*	11 ***3***	NR	NR	NR	NR
PS 0	207 ***33***	199 ***32***	187 ***29***	189 ***29***	27 ***20***	37 ***27***	84 ***29***	95 ***33***	251 ***88.6***	252 ***87.6***	112 ***33***	116 ***34***	46 ***32***	45 ***32***	52 ***48***	53 ***48***	155 ***36***	160 ***38***	126 ***32***	134 ***34***	37 ***22.3***	44 ***26.5***
PS 1	420 ***67***	425 ***68***	467 ***71***	470 ***71***	106 ***79***	100 ***73***	208 ***71***	194 ***67***			229 ***67***	224 ***65***	96 ***68***	97 ***68***	48 ***44***	50 ***45***	270 ***64***	265 ***62***	269 ***68***	262 ***66***	98 ***59***	104 ***62.7***
Current & former smoker	518 ***82***	483 ***77***	490 ***75***	498 ***76***	121 ***90***	129 ***94***	231 ***79***	227 ***78***	NA	NA	279 ***81***	269 ***78***	117 ***81***	114 ***80***	90 ***83***	80 ***73***	341 ***80***	353 ***83***	361 ***91***	367 ***92***	128 ***77.1***	124 ***74.7***
Never smoker	109 ***17***	141 ***23***	165 ***25***	161 ***24***	10 ***7***	7 ***5***	58 ***20***	60 ***21***			63 ***18***	67 ***20***	27 ***19***	29 ***20***	19 ***17***	30 ***27***	84 ***20***	72 ***17***	26 ***7***	18 ***5***	24 ***14.5***	29 ***17.5***
Stage IIIB at inclusion	0	0	148 ***23***	146 ***22***	29 ***21***	24 ***18***	20 ***7***	24 ***8***	71 ***25.1***	73 ***25.3***	na	na	NR	NR	NR	NR	NR	NR	48 ***12***	48 ***12***	19 ***11.4***	12 ***7.2***
Stage IV at inclusion	628,***100***	625,***100***	399 ***61***	408 ***62***	105 ***78***	112 ***82***	272 ***93***	266 ***92***	212 ***74.9***	215 ***74.7***	na	na	NR	NR	NR	NR	NR	NR	349 ***88***	345 ***87***	147 ***88.6***	154 ***92.8***
Non-squamous	465 ***74***	447 ***72***	347 ***53***	352 ***53***	0	0	292,***100***	290,***100***	154 ***54.4***	142 ***49.3***	240 ***70***	240 ***70***	95 ***66***	95 ***66***	78 ***71.5***	87 ***79***	313 ***74***	315 ***74***	17 ***4***	15 ***4***	130 ***79.3***	127 ***76.5***
Squamous	157 ***25***	171 ***27***	276 ***42***	279 ***42***	135,***100***	137,***100***	0	0	78 ***27.6***	93 ***32.3***	76 ***22***	66 ***19***	49 ***34***	48 ***34***	31 ***28.4***	23 ***21***	112 ***26***	110 ***26***	381 ***96***	382 ***96***	36 ***21.7***	39 ***23.5***
Prior platinum-based therapy	623 ***99***	622 ***99***	628 ***97***	636 ***98***	135,***100***	138,***100***	292,***100***	290,***100***	262 ***92.6***	259 ***89.9***	na	na	NR	NR	109,***100***	110,***100***	425,***100***	425,***100***	398,***100***	397,***100***	166,***100***	166,***100***
First-line bevacizumab	88 ***14***	92 ***15***	27 ***4***	23 ***4***	1 ***1***	2 ***1***	na	na	0	0	na	na	NR	NR	NR	NR	NR	NR	NR	NR	NR	NR
Prior maintenance treatment	135 ***21***	143 ***23***	NA	NA	NA	NA	122 ***42***	111 ***38***	NA	NA	na	na	144,***100***	143,***100***	109,***100***	109 ***99***	NR	NR	NR		NR	NR
Previous taxane	153 ***24***	152 ***24***	NA	NA	46 ***34***	46 ***34***	na	na	73 ***25.8***	80 ***27.8***	na	na	NR	NR	0	0	NR	NR	NR	NR	NR	NR
EGFR Wild type	207 ***33***	197 ***32***	NA	NA	NA	NA	na	na	NA	NA	293 ***85***	294 ***86***	NR	NR	109,***100***	110,***100***	318 ***75***	310 ***73***	NR	NR	57 / 62	55 / 61
EGFR Mutant	15 ***2***	18 ***3***	NA	NA	NA	NA	44 ***15***	38 ***13***			28 ***8***	26 ***8***	10 ***12***	8 ***10***	0	0	42 ***10***	43 ***10***	NR	NR	5 / 62	6 / 61
Unknown or missing	406 ***65***	410 ***66***	NA	NA	NA	NA	na	na			23 ***7***	23 ***7***	NR	NR	0	0	65 ***15***	72 ***17***	NR	NR	NR	NR
1 prior therapy	628,***100***	625,***100***	655,***100***	659,***100***	135,***100***	137,***100***	292,***100***	290,***100***	283,***100***	288,***100***	243 ***71***	235 ***69***	93 ***65***	96 ***67***	unclear	unclear	320 ***75***	320 ***75***	398,***100***	397,***100***	101 ***60.8***	89 ***53.6***
2 prior therapies	0	0	0	0	0	0	0	0	0	0	66 ***19***	75 ***22***	51 ***35***	47 ***33***			105 ***25***	105 ***25***	0	0	65 ***39.2***	77 ***46.4***

Bold italicised numbers refer to a range or a percentage

Nine studies [15–18, 20–22, 24, 26] were considered as high-risk of bias due to the lack of blinding of participants and personnel. The five RCTs [15–17, 21, 24] evaluating checkpoint inhibitors versus DOC were open-label and were considered as high-risk due to performance bias. LUME-LUNG-1 [23] was rated at low risk of bias for all the key domains. Only HORG and TAILOR [18, 22] had public funding, so the remaining studies were rated as high-risk due to “other source bias”.

### Overall survival analyses in mixed histology populations

These analyses were based on mixed populations of patients whose tumour histology was either squamous or non-squamous.

#### Overall survival from observed data

**Fig. 1:**
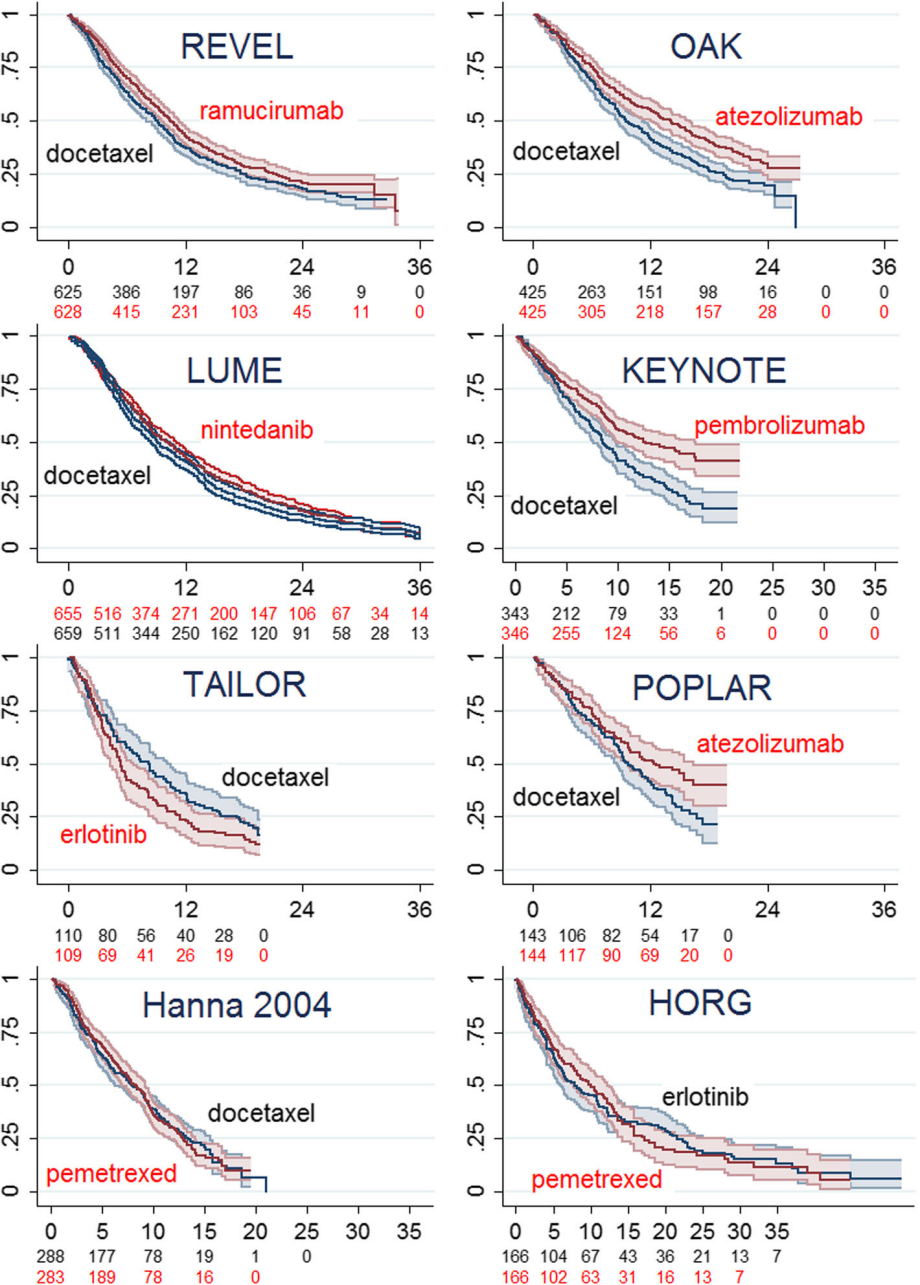
Reconstructed Kaplan-Meier plots (95% CI) of overall survival; studies recruiting patients irrespective of tumour histology. Time axis is months, vertical axis is proportion alive

Reconstructed KM plots from studies reporting OS in populations unselected according to tumour histology are shown in Fig. 1 (for completeness of analysis corresponding plots for PFS are presented in Additional file 4). Only the plots for ATEZO (OAK [24] and POPLAR [17] trials) and for PEMBRO (KEYNOTE-010 [21]) imply appreciable survival gains over DOC. ERLO was not beneficial compared to DOC (TAILOR [18]) or PEM (HORG trial, [22]).

RMS estimates are summarised in Additional file 5. Over the observed period of 19 months common to all studies of new treatments, the RMS delivered by DOC (alone or combined with placebo) ranged between 9.30 (95% CI 8.02–10.57) months (TAILOR) and 10.68 (95% CI 10.03–11.33) months (OAK), while in the older study of DOC vs. PEM (Hanna et al., 2004 [20], post hoc analysis by Scagliotti et al. 2009 [25]) DOC delivered only 8.70 months (95% CI 7.96–9.44) RMS (Table 2). The RMS gain relative to DOC from new drugs over 19 months was modest ranging from minus 1.64 months (95% CI minus 3.36–0.08) for ERLO, 0.48 months (95% CI minus 0.23–1.18) and 0.99 months (95% CI 0.24–1.73) for NIN + DOC and RAM+DOC respectively, to between 1.45 months (95% CI minus 0.11–3.00) and 1.62 months (95% CI 0.70–2.55) for ATEZO and 1.58 months (95% CI 0.48–2.68) for PEMBRO (Table 1 and Additional file 6).

**Table 2:** Mean survival (months) estimates from studies of patients with mixed histologies

TRIAL	Outcome	Intervention (*n*)	Control (*n*)	Intervention minus control
REVEL		Ram + Doc (628)	Plac + Doc (625)	
RMS [95% CI]	to 19 mos	11.00 [10.47–11.52]	10.01 [9.48–10.55]	0.99 [0.24–1.73]
Mean total OS	*R_mSext*	15.02	14.31	0.71
Mean total OS	Weibull [95% CI]	14.87 [13.40–16.57]	12.99 [11.71–14.46]	1.88 [−0.22–3.98]
Mean total OS	Weibull formula	14.87	12.98	1.89
LUME LUNG-1		Nin + Doc (655)	Plac + Doc (659)	
RMS [95% CI]	to 19 mos	10.85 [10.35–11.36]	10.38 [9.88–10.87]	0.48 [−0.23–1.18]
Mean total OS	*R_mSext*	14.38	13.57	0.82
Mean total OS	Weibull [95% CI]	14.08 [12.97–15.31]	13.21 [12.17–14.35]	0.87 [− 0.73–2.47]
Mean total OS	Weibull formula	14.08	13.20	0.88
POPLAR		Atezolizumab (144)	Docetaxel (143)	
RMS [95% CI]	to 19 mos	11.84 [10.71–12.97]	10.39 [9.33–11.46]	1.45 [−0.11–3.00]
Mean total OS	*R_mSext*	20.76	13.00	7.76
Mean total OS	Weibull [95% CI]	17.89 [13.69–24.31]	12.15 [10.02–15.05]	5.74 [−0.135–11.61]
Mean total OS	Weibull formula	17.93	12.15	5.78
OAK		Atezolizumab (425)	Docetaxel (425)	
RMS [95% CI]	to 19 mos	12.31 [11.65–12.96]	10.68 [10.03–11.33]	1.62 [0.70–2.55]
Mean total OS	*R_mSext*	20.76	12.24	8.52
Mean total OS	Weibull [95% CI]	18.93 [16.54–21.81]	13.59 [12.11–15.32]	5.34 [2.25–8.43]
Mean total OS	Weibull formula	18.98	13.34	5.64
KEYNOTE-010		Pembrolizumab (344)	Docetaxel (343)	
RMS [95% CI]	to 19 mos	11.40 [10.62–12.19]	9.82 [9.05–10.59]	1.58 [0.48–2.68]
Mean total OS	*R_mSext*	20.64	12.74	7.89
Mean total OS	Weibull [95% CI]	16.14 [13.51–19.68]	11.10 [9.68–12.88]	5.04 [1.57–8.52]
Mean total OS	Weibull formula	16.43	10.42	6.01
TAILOR		Erlotinib (109)	Docetaxel (110)	
RMS [95% CI]	to 19 mos	7.66 [6.15–8.81]	9.30 [8.02–10.57]	−1.64 [−3.36–0.08]
Mean total OS	*R_mSext*	8.90	11.16	−2.26
Mean total OS	Weibull [95% CI]	8.67 [6.99–10.86]	11.11 [8.80–14.25]	− 2.44 [−5.78–0.90]
Mean total OS	Weibull formula	8.67	11.10	−2.43
HORG		Erlotinib (166)	Pemetrexed (166)	
RMS [95% CI]	to 19 mos	10.18 [9.10–11.26]	9.85 [8.73–10.97]	0.33 [− 1.23–1.88]
Mean total OS	*R_mSext*	15.33	14.42	0.91
Mean total OS	Weibull [95% CI]	15.02 [11.94–18.94]	13.86 [11.21–17.35]	1.16 [−3.5–5.82]
Mean total OS	Weibull formula	15.12	13.86	1.25
Hanna		Pemetrexed (283)	Docetaxel (288)	
RMS [95% CI]	to 19 mos	8.80 [8.10–9.50]	8.70 [7.96–9.44]	0.10 [−0.92–1.12]
Mean total OS	*R_mSext*	9.64	8.83	0.81
Mean total OS	Weibull [95% CI]	9.34 [8.30–10.57]	9.35 [8.20–10.74]	−0.01 [−1.71–1.69]
Mean total OS	Weibull formula	9.34	9.34	−0.01

*OS* overall survival, *RMS* restricted mean survival; *R_mSext* restricted mean survival exponentially extended from the end of the KM plot, *Mean total OS Weibull formula* mean OS estimated from Weibull model parameters using the formula published by Davies et al. [13]

#### Overall survival from extrapolated data (survival modelling)

**Fig. 2:**
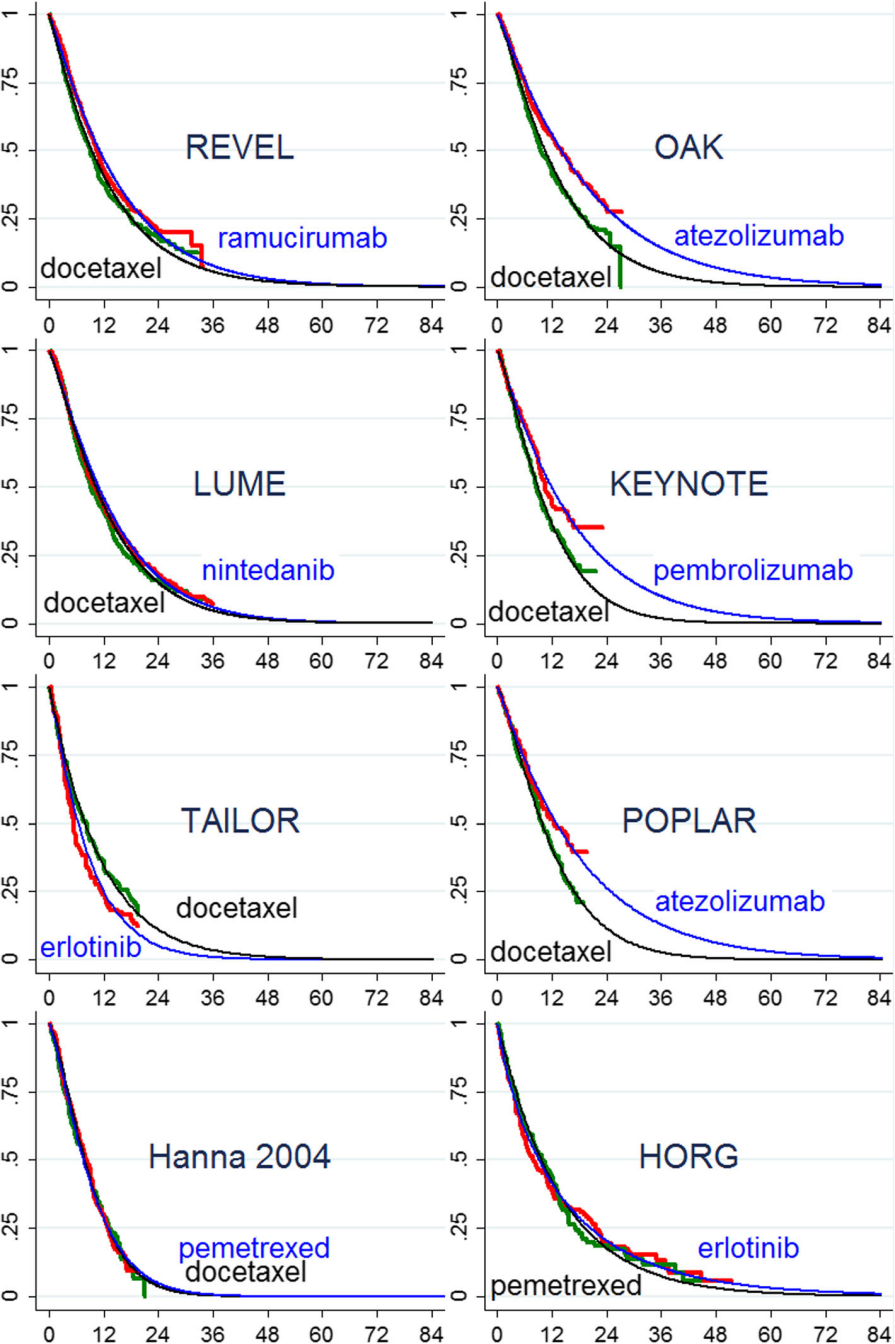
Weibull models of overall survival for studies depicted in Fig. 1. Time axis is months, vertical axis is proportion alive

**Fig. 3:**
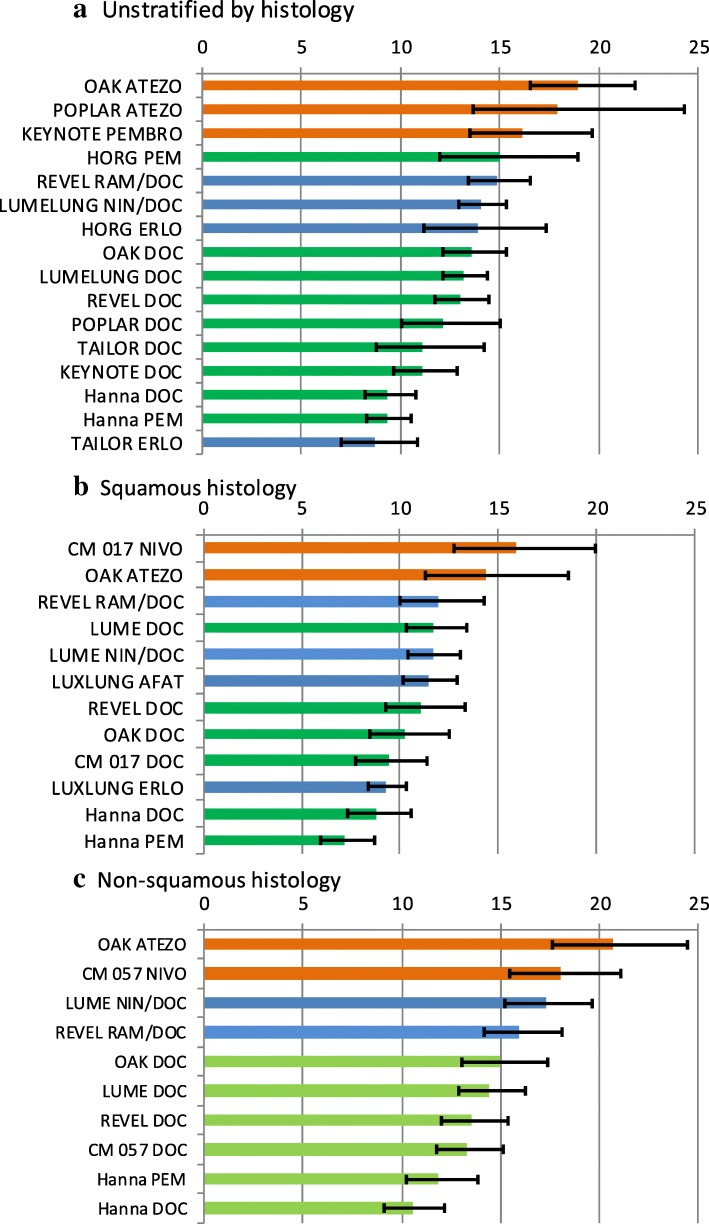
Compared mean survivals (calculated using Weibull model) irrespective of histology (**a**), in squamous histology (**b**), and non-squamous histology (**c**); orange bars denote immunotherapies, blue bars targeted therapies, and green bars chemotherapies

Exponential extrapolation from the tail of the KM plots (Stata command: *stci, emean*) suggests losses for ERLO relative to DOC, and gains over DOC of less than 1 month for RAM+DOC and NIN + DOC (the latter licensed only for adenocarcinoma), and potentially impressive gains over DOC of 7.9 to 8.5 months for PEMBRO and ATEZO respectively (Table 2). However, the alternative procedure of modelling OS using Weibull fits to the whole of the KM plot suggests more modest gains for immunotherapies relative to DOC (Fig. 2 and Table 2). Across industry-sponsored studies of immuno- and targeted therapies Weibull models of overall survival (Fig. 3) with DOC yielded between 11.10 months (95% CI: 9.98–12.88) (KEYNOTE-010 [21]) and 13.59 months (95% CI: 12.11–15.32) (OAK), and suggest mean survival gains over DOC of 5.74 months (95% CI minus 0.14–11.61) and 5.34 months (95% CI 2.25–8.43) for ATEZO (POPLAR [17] and OAK [24] respectively), 5.04 months (95% CI 1.57–8.52) for PEMBRO (KEYNOTE-010), but of less than 2 months for targeted therapies RAM+DOC (REVEL [19]) and NIN + DOC (LUME LUNG-1 [23]), and no gain for ERLO. Weibull modelling of the publicly funded HORG trial indicated a possible modest gain from ERLO over PEM (1.16 months; 95% CI: minus 3.5–5.82), and modelling of the Hanna study indicated likely equivalence of the chemotherapies DOC and PEM.

### Overall survival analyses per histology (squamous or non-squamous)

These analyses were based on the studies where KM plots for trial participants stratified according to histology were presented.

#### Mean survival from observed data

**Fig. 4:**
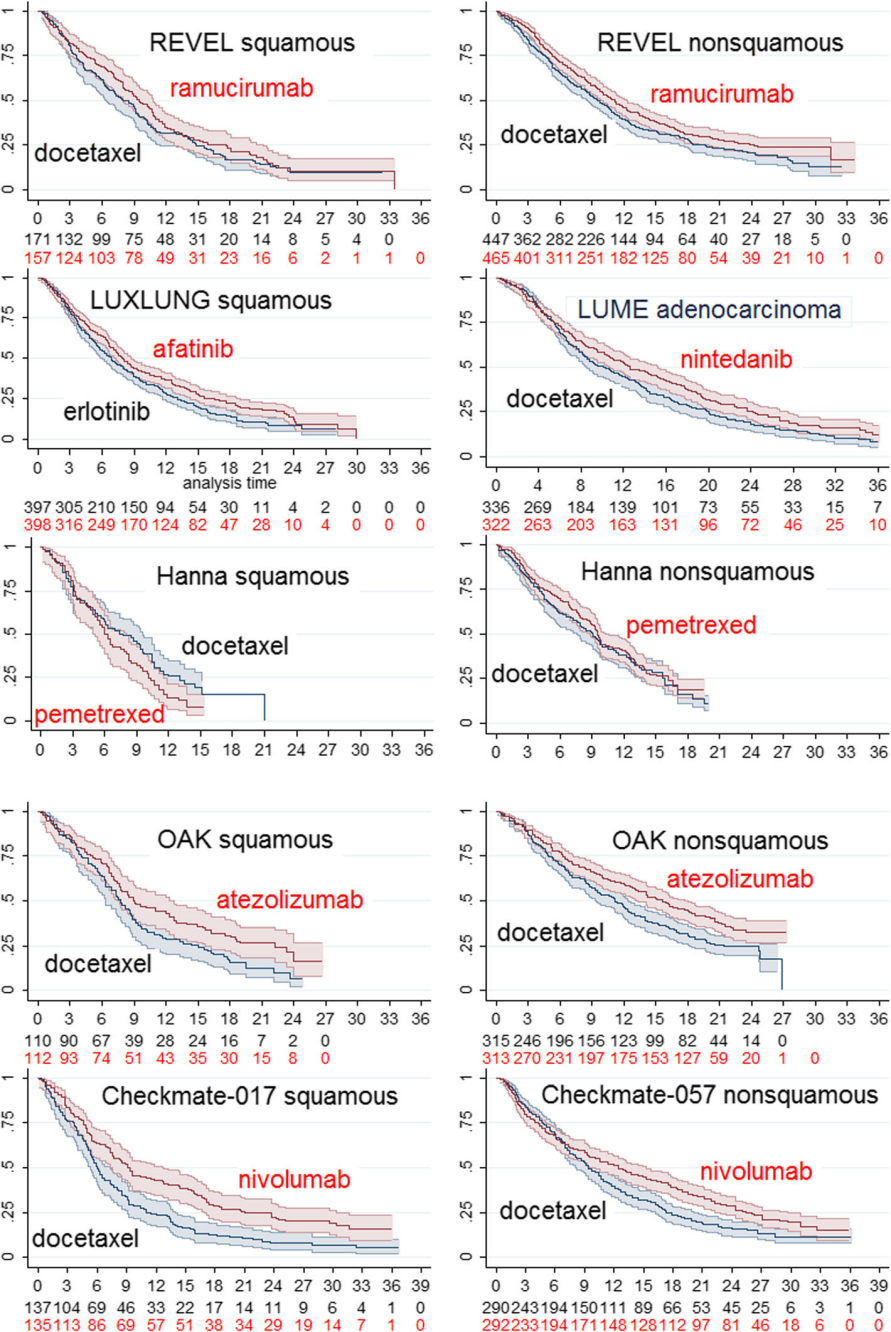
Reconstructed Kaplan-Meier (95% CI) plots of overall survival; studies recruiting patients with specified tumour histology. Time axis is months, vertical axis is proportion alive

Figure 4 summarises the reconstructed KM plots for licenced drugs for squamous histology and non-squamous histology. These suggest likely modest gains from RAM+DOC irrespective of histology and for NIN + DOC in the treatment of adenocarcinoma (the licensed indication), little or no gain from PEM over DOC irrespective of histology, but more substantial likely gains over DOC from the checkpoint inhibitors (NIVO and ATEZO) for both histology types. No KM plots per histology were available for PEMBRO.

Over the observed periods of 24 and 27 months common to all squamous and non-squamous studies respectively, NIVO and ATEZO delivered between about 2 and 4 months RMS gain over DOC, while RAM+DOC and NIN + DOC only between 1 and 2 months, results supporting the apparent superiority of the checkpoint inhibitors (Tables 3 and 4, Additional file 6).

**Table 3:** Estimates of mean survival (months) based on studies of patients with squamous histology

TRIAL	Outcome	Intervention (n)	Control (n)	Intervention minus control
REVEL		Ram + Doc (157)	Plac + Doc (171)	
RMS [95% CI]	to 24 mos	10.89 [19.65–12.13]	9.92 [8.75–11.10]	0.96 [− 0.75–2.67]
Mean total OS	*R_mSext*	12.04	11.87	0.17
Mean total OS	Weibull [95% CI]	11.91 [10.01–14.29]	11.08 [9.31–13.29]	0.83 [−2.09–3.75]
Mean total OS	Weibull formula	11.90	11.07	0.83
Lux-lung 8		Afatanib (398)	Erlotinib (397)	
RMS [95% CI]	to 24 mos	10.48 [9.67–11.28]	8.95 [8.23–9.67]	1.52 [0.44–2.61]
Mean total OS	*R_mSext*	10.98	9.87	1.11
Mean total OS	Weibull [95% CI]	11.46 [10.19–12.94]	9.32 [8.39–10.37]	2.14 [0.45–3.83]
Mean total OS	Weibull formula	11.35	9.41	1.94
LUME LUNG-1		Nin + Doc (276)	Docetaxel (279)	
RMS [95% CI]	to 24 mos	10.65 [9.79–11.52]	10.14 [9.26–11.02]	0.51 [−0.72–1.75]
Mean total OS	*R_mSext*	11.76	12.19	−0.43
Mean total OS	Weibull [95% CI]	11.67 [10.42–13.07]	11.73 [10.31–13.38]	−0.06 [−2.09–1.97]
Mean total OS	Weibull formula	11.67	11.72	−0.06
Checkmate_017		Nivolumab (135)	Docetaxel (137)	
RMS [95% CI]	to 24 mos	11.94 [10.48–13.39]	8.33 [7.15–9.52]	3.61 [1.73–5.48]
Mean total OS	*R_mSext*	17.14	9.76	7.37
Mean total OS	Weibull [95% CI]	15.92 [12.79–19.94]	9.41 [7.78–11.41]	6.51 [2.50–10.52]
Mean total OS	Weibull formula	15.95	9.40	6.55
OAK		Atezolizumab (112)	Docetaxel (110)	
RMS [95% CI]	to 24 mos	11.99 [10.37–13.62]	9.73 [8.31–11.14]	2.27 [0.11–4.42]
Mean total OS	*R_mSext*	14.80	10.41	4.40
Mean total OS	Weibull [95% CI]	14.34 [11.31–18.58]	10.26 [8.45–12.52]	4.08 [−0.09–8.25]
Mean total OS	Weibull formula	14.34	10.25	4.09
Hanna		Pemetrexed (78)	Docetaxel (94)	
RMS [95% CI]	to 24 mos	NOT REACHED		
Mean total OS	*R_mSext*	7.40	8.83	−1.43 [−0.75–2.67]
Mean total OS	Weibull [95% CI]	7.22 [5.95–8.75]	8.83 [7.32–10.59]	− 1.61 [−5.84–2.62]
Mean total OS	Weibull formula	7.22	8.82	−1.61

*OS* overall survival, *RMS* restricted mean survival, *R_mSext* restricted mean survival exponentially extended from the end of the KM plot, *Mean total OS Weibull formula* mean OS estimated from Weibull model parameters using the formula published by Davies et al. [13]

**Table 4:** Estimates of mean survival (months) based on studies of patients with non- squamous histology

TRIAL	Outcome	Intervention (*n*)	Control (*n*)	Intervention minus control
REVEL		Ram + Doc (465)	Plac + Doc (447)	
RMS [95% CI]	to 27 mos	13.50 [12.60–14.40]	12.10 [11.20–13.00]	1.39 [0.12–2.67]
Mean total OS	*R_mSext*	18.18	14.88	3.31
Mean total OS	Weibull [95% CI]	15.98 [14.16–18.15]	13.56 [12.00–15.41]	2.42 [−0.20–5.04]
Mean total OS	Weibull formula	16.98	13.56	2.43
LUME LUNG-1		Nin + Doc (322)	Plac + Doc (336)	
RMS [95% CI]	to 27 mos	14.18 [13.14–15.21]	12.62 [11.65–13.59]	1.55 [0.14–2.97]
Mean total OS	*R_mSext*	17.84	14.90	2.94
Mean total OS	Weibull [95% CI]	17.29 [15.24–19.68]	14.45 [12.88–16.26]	2.84 [0.05–5.63]
Mean total OS	Weibull formula	17.30	14.45	2.85
Checkmate_057		Nivolumab (292)	Docetaxel (290)	
RMS [95% CI]	to 27 mos	13.93 [12.77–15.09]	11.79 [10.78–12.80]	2.14 [0.61–3.68]
Mean total OS	*R_mSext*	18.29	14.72	3.57
Mean total OS	Weibull [95% CI]	18.04 [15.48–21.07]	13.32 [11.73–15.18]	4.72 [1.44–8.00]
Mean total OS	Weibull formula	18.13	13.31	4.82
OAK		Atezolizumab (313)	Docetaxel (315)	
RMS [95% CI]	to 27 mos	15.62 [14.5–16.72]	13.07 [11.99–14.15]	2.55 [1.00–4.09]
Mean total OS	*R_mSext*	23.76	13.09	10.67
Mean total OS	Weibull [95% CI]	20.70 [17.64–24.51]	15.02 [13.05–17.43]	5.68 [1.61–9.75]
Mean total OS	Weibull formula	20.79	15.01	5.77
Hanna		Pemetrexed (205)	Docetaxel (194)	
RMS [95% CI]	to 27 mos	na	na	na
Mean total OS	*R_mSext*	12.54	10.72	1.82
Mean total OS	Weibull [95% CI]	11.88 [10.27–13.82]	10.53 [9.11–12.20]	1.35 [−1.00–3.70]
Mean total OS	Weibull formula	11.87	10.52	1.35

*OS* overall survival, *RMS* restricted mean survival, *R_mSext* restricted mean survival exponentially extended from the end of the KM plot, *Mean total OS Weibull formula* mean OS estimated from Weibull model parameters using the formula published by Davies et al. [13]

#### Overall survival from extrapolated data (survival modelling)

**Fig. 5:**
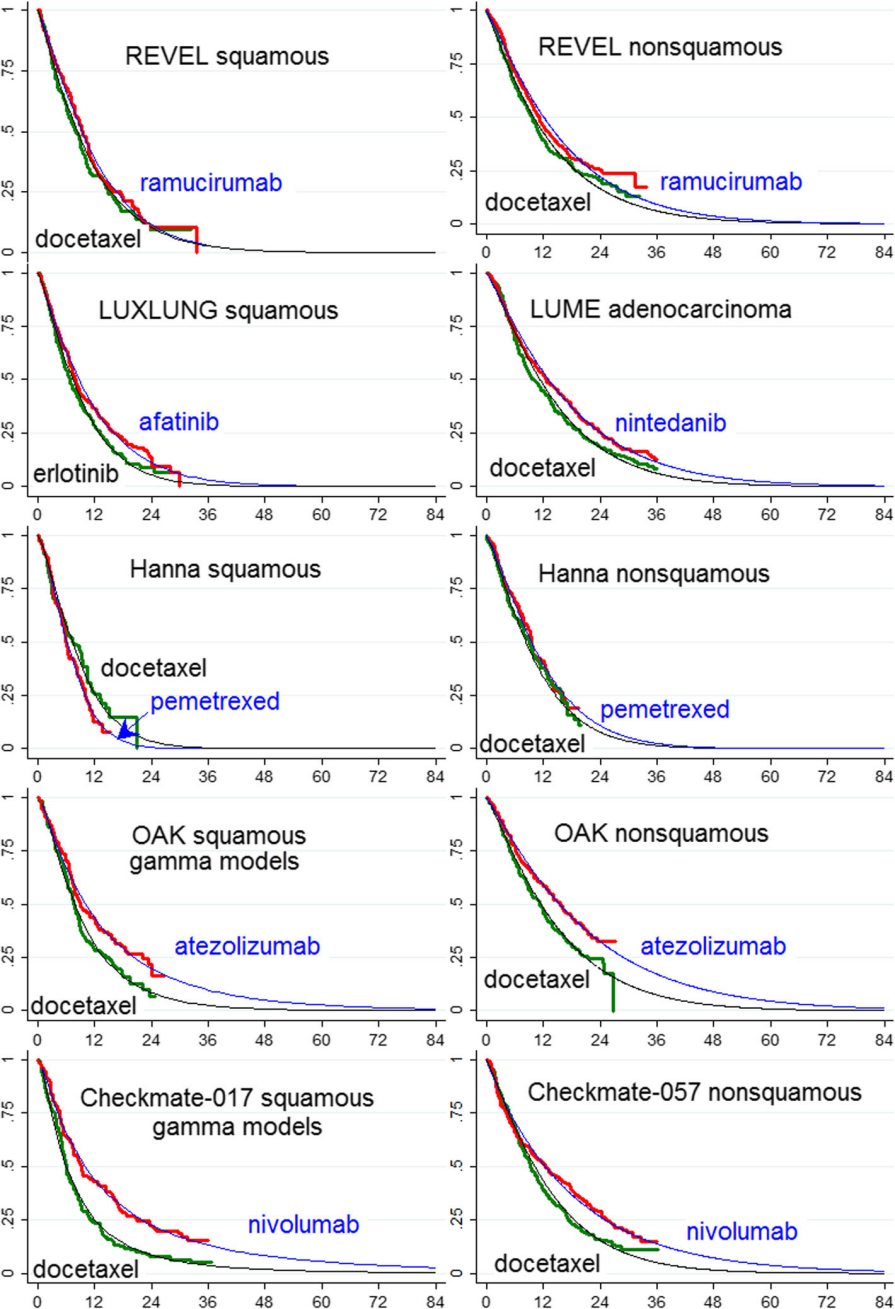
Parametric models of overall survival for studies depicted in Fig. 3. Time axis is months, vertical axis is proportion alive. All are Weibull models except where specified

Weibull models provided satisfactory fits for non-squamous histology but the shapes of the KM plots for squamous histology for the checkpoint agents were irregular and gamma models provided a better fit. Parametric models are summarised in Fig. 5.

For the industry-sponsored studies of targeted and immunotherapies Weibull model estimates of mean survival with DOC treatment in patients with squamous histology ranged between 9.41 (95% CI: 7.78–11.41) months (CHECKMATE-017) and 11.73 (95% CI: 10.131–13.38) months (LUME LUNG-1), and in patients with non-squamous histology between 13.32 (95% CI: 11.73–15.8) months (CHECKMATE-057) and 15.02 (95% CI: 13.05–17.43) months (OAK) (Tables 3 and 4, and Fig. 3). The gain in overall mean survival over DOC from targeted and immunotherapies therapies for patients with squamous histology ranged from less than 1 month for RAM+DOC and NIN + DOC to 4.08 months (95% CI minus 0.09–8.25) for ATEZO and 6.51 months (95% CI 2.50–10.52) for NIVO (CHECKMATE-017) (Tables 3 and 4, Additional file 6). Mean survival gains over DOC of 4.81 months for ATEZO and 7.45 months for NIVO were obtained if better fitting gamma models were substituted for Weibull models for squamous histology (Fig. 5), while gamma models for targeted therapies yielded smaller gains than Weibull models. Survival gain from AFA over ERLO was estimated to be 2.14 months (95% CI 0.45–3.83) (the reported HR for OS was 0.82); this gain would probably diminish if the comparator had been DOC since the TAILOR trial [18] found superior performance for DOC over ERLO in both squamous and non-squamous histology populations (HR 1.11, 95% CI: 0.61–2.03, and 1.49 95% CI: 1.06–2.10, respectively), however AFA might be expected to have a superior safety profile to DOC. The mean gain in survival over DOC from targeted and immunotherapies therapies for patients with non-squamous histology (Table 4) ranged between 2.42 months (95% CI minus 0.20–5.04) and 2.84 months (95% CI minus 0.05–5.63) for RAM+DOC and NIN + DOC, to 4.72 months (95% CI minus 1.44–8.00) and 5.68 months (95% CI minus 1.61–9.75) for ATEZO and NIVO respectively.

In network analysis immunotherapies consistently ranked higher than alternatives irrespective of population histology and outcome measure (Additional file 7).

### Exploratory analyses on PFS and OS relationships

PFS is often specified as a primary or co-primary outcome in trials of cancer drugs. We conducted analyses to explore if PFS in NSCLC might be an indicator for overall survival in second line therapies. Weibull model estimates of gains in PFS over DOC for targeted therapies and immunotherapies were modest ranging from + 1.18 months (RAM + DOC) to minus 1.33 months (ERLO) in studies recruiting patients unrestricted by histology (Additional file 4). Available data for squamous and non-squamous histologies indicated similarly small gains except in the case of CHECKMATE-017 (squamous histology) in which the estimated gain was more substantial (3.11 months). Across the included studies there was a poor relationship between modelled estimates of PFS and of OS, and between modelled PFS gains and reported PFS hazard ratios, whereas strong associations were seen between modelled OS and reported median OS, and between modelled OS gains and reported OS hazard ratios (Additional file 8). These finding suggests that PFS is unlikely to be a good indicator for subsequent OS in this case.

## Discussion

In this study we estimated the mean number of months of survival benefit from therapies licensed for the treatment of advanced NSCLC. An estimation of survival in the absence of treatment can be obtained from two early RCTs in NSCLC patients, previously treated with platinum chemotherapy, and who were randomised to receive placebo or best supportive care (BSC). The reported median survivals were 4.7 [27] and 4.6 months (95% CI: 3.7–6.0) [28] respectively. By applying the methods described above using Weibull models, we estimate BSC and placebo mean survival to be 7.34 months (95% CI: 5.92–9.14) and 7.77 months (95% CI: 6.71–9.03). If patients received DOC they might expect an extension in average life expectancy to about a year depending on histology, with slightly better prospects for those with non-squamous histology.

Our results suggest that mean survival gains over DOC from RAM+DOC, NIN + DOC, and ERLO, are meagre but may be marginally superior for patients with non-squamous than for those with squamous tumour histology. The analysed results indicate that average survival gains over DOC from checkpoint inhibitors are greater than those from the targeted therapies, with estimates for the former reaching between 4 and 9 months depending on tumour histology and the method of modelling beyond the observed data.

The European Society for Medical Oncology (ESMO) has recognised that a comparison of treatments based solely on hazard ratios for OS provide only indirect information about treatment benefit; they have proposed an estimator, the “Magnitude of Clinical Benefit Scale (ESMO-MCBS)”, which they believe represents “*a standardised, generic, validated approach to stratify the magnitude of clinical benefit that can be anticipated from anti-cancer therapies*” [29]. Davis et al. 2017 [4] used this tool to examine pharmaceutical interventions for advanced cancers approved by the EMA 2009 to 2013. The authors expressed concern that for many of these interventions, the available evidence failed to demonstrate survival benefit or improved patient quality of life.

Patients may have difficulty in interpreting measures of relative risk (e.g. HRs) and in understanding the basis of the ESMO-MCBS tool measure. Patients often prefer information about the likely lifetime gain (e.g. life years gained) from a new treatment being offered. It has been suggested that advanced cancer patients with short life expectancy are willing to accept considerable toxicity of treatments that offer a chance of durable survival [30], however evidence on this is conflicting [31]. It has been claimed that a proportion of patients who receive immunotherapies may experience a durable survival response (a so called “tail of the curve response”) so that mean survival estimates for the “whole population” may mask this possibility. However, the evidence base for such outcomes is far from clear cut.

The British Thoracic Society has provided guidance for health care professionals about sharing information with patients with lung cancer. [32] Such information could include estimates of average survival benefit that might accrue with various treatment options. Furthermore decision makers such as NICE generally require estimates of the mean gain in survival from new treatments when taking reimbursement decisions. It is therefore of interest to gain an idea of the mean survival benefit yielded from new treatments for advanced NSCLC and to see how such benefit might vary according to tumour histology.

Equally importantly, mean survival gains offer an unambiguous, informative measure of outcome, which is far less exposed to limitations and controversies surrounding the use of quality-adjusted life years (QALYs) in the evaluation of treatments for neoplasms. While the QALYs facilitates decision making across areas, limitations in the way QALYs are constructed have led to criticism on various grounds (including insensitivity to changes in health states [33], especially those that are caused by adverse effects due to cancer treatments [34]. Limitations in QALYs have led researcher to conclude that ‘*the measure shows important limitations in terms of its ability to accurately capture the value of the health gains deemed important by cancer patients’* [33]. Reimbursement decisions become challenging when comparing cancer, with its’ generally short term survival expectation, with chronic disabling diseases with relatively extended absolute survival. Given this, we expect that estimates of survival are key information which, at the very least, should be reported and considered alongside QALYs.

Our review has several strengths. To the best of our knowledge, this is the first attempt at comparing mean survival of all drugs with a licensed indication for second/third line treatment of advanced/metastatic wild-type NSCLC. It is justified, because the growing number of licensed therapies offers a new range of treatment options for which survival information is of interest to both oncologists and patients. Multi-arm RCTs could provide the best evidence, but these have not been undertaken and our work provides a pragmatic approach.

Our review has several limitations. Although we used rigorous methods to identify all relevant literature we could only include 11 primary research studies so the inherent risk of publication bias may be of particular importance. Our survival curves and estimates have relied on reconstructing the underlying individual patient data rather than using the individual patient data itself. However for all the included studies, there was a close correspondence between our derived curves and those published. A further potential limitation is the risk of uneven performance of the common comparator, DOC, between different studies; however these differences were small relative to differences between targeted therapies and the checkpoint inhibitors. We noted some differences in baseline characteristics across studies regarding the number of prior lines of treatment and disease stage at inclusion. For these variables survival outcomes were not reported in sufficient detail to allow sensitivity analyses to test the robustness of our results.

## Conclusion

Based on our review, NIVO, PEMBRO and ATEZO exhibit superior benefit compared to other licensed drugs indicated for people with non-specific late stage NSCLC. The patient survival gains over chemotherapy from these drugs appear to be fairly substantial in the context of an expected average survival with DOC of less than 1 year for people with squamous histology and a little over a year for those with non-squamous histology.

## Additional files

Additional file 1: Medline search strategy. (DOCX 13 kb)

Additional file 2: PRISMA study flow chart (DOCX 28 kb)

Additional file 3: Reconstructed hazard ratios compared to published hazard ratios. (DOCX 19 kb)

Additional file 4: Results for Progression Free Survival (PFS). (DOCX 4601 kb)

Additional file 5: Restricted mean survival (RMS) results (DOCX 28 kb)

Additional file 6: Forest plots of mean difference in RMS and mean difference in total survival (Weibull models). (DOCX 43 kb)

Additional file 7: Network meta-analysis estimating mean differences in restricted mean survival and overall survival (DOCX 26 kb)

Additional file 8: Association of survival estimates and reported medians and hazard ratios. (DOCX 32 kb)

## Abbreviations

ALK: Anaplastic lymphoma kinase
ATEZO: Atezolizumab
CHPM: Committee of Medicinal Products for Human use
DOC: Docetaxel
EGFR: Epidermal Growth Factor Receptor
EMA: European Medicines Agency
ERL: Erlotinib
IPD: Individual patient data
NIN: Nintedanib
NIVO: Nivolumab
NSCLC: Non-small cell lung cancer
OS: Overall survival
PEM: Pemetrexed
PEMBRO: Pembrolizumab
PFS: Progression free survival
PRISMA: Preferree reporting items for Systematic Reviews and Meta-analyses
RAM: Ramucirumab
RoB: Risk of bias

## References

[1] Novello S, Barlesi F, Califano R, Cufer T, Ekman S, Levra MG, Kerr K, Popat S, Reck M, Senan S (2016). Metastatic non-small-cell lung cancer: ESMO clinical practice guidelines for diagnosis, treatment and follow-up. Ann Oncol.

[2] Royal College of Physicians. National Lung Cancer Audit 2015 [https://www.rcplondon.ac.uk/projects/national-lung-cancer-audit Accessed 27 Feb 2019].

[3] Clegg A, Scott DA, Sidhu M, Hewitson P, Waugh N (2001). A rapid and systematic review of the clinical effectiveness and cost-effectiveness of paclitaxel, docetaxel, gemcitabine and vinorelbine in non-small-cell lung cancer. Health Technol Assess.

[4] Davis C, Naci H, Gurpinar E, Poplavska E, Pinto A, Aggarwal A (2017). Availability of evidence of benefits on overall survival and quality of life of cancer drugs approved by European medicines agency: retrospective cohort study of drug approvals 2009-13. BMJ (Clinical research ed).

[5] Armoiry X, Tsertsvadze A, Connock M, Royle P, Melendez-Torres GJ, Souquet PJ, Clarke A (2018). Comparative efficacy and safety of licensed treatments for previously treated non-small cell lung cancer: a systematic review and network meta-analysis. PLoS One.

[6] Crequit P, Chaimani A, Yavchitz A, Attiche N, Cadranel J, Trinquart L, Ravaud P (2017). Comparative efficacy and safety of second-line treatments for advanced non-small cell lung cancer with wild-type or unknown status for epidermal growth factor receptor: a systematic review and network meta-analysis. BMC Med.

[7] Moher D, Shamseer L, Clarke M, Ghersi D, Liberati A, Petticrew M, Shekelle P, Stewart LA (2015). Preferred reporting items for systematic review and meta-analysis protocols (PRISMA-P) 2015 statement. Syst Rev.

[8] Higgins JP, Altman DG, Gotzsche PC, Juni P, Moher D, Oxman AD, Savovic J, Schulz KF, Weeks L, Sterne JA (2011). The Cochrane Collaboration's tool for assessing risk of bias in randomised trials. BMJ.

[9] Guyot P, Ades AE, Ouwens MJ, Welton NJ (2012). Enhanced secondary analysis of survival data: reconstructing the data from published Kaplan-Meier survival curves. BMC Med Res Methodol.

[10] Royston P, Parmar MK (2013). Restricted mean survival time: an alternative to the hazard ratio for the design and analysis of randomized trials with a time-to-event outcome. BMC Med Res Methodol.

[11] Cronin A, Tian L, Uno H (2016). strmst2 and strmst2pw: new commands to compare survival curves using the restricted mean survival time. Stata J.

[12] Crowther M, Lambert P. Stgenreg: a Stata package for the general parametric analysis of survival data. J Stat Softw. 2013;53(12):1–17.

[13] Davies A, Briggs A, Schneider J, Levy A, Ebeid O, Wagner S, Kotapati S, Ramsey S (2012). The ends justify the mean: outcome measures for estimating the value of new Cancer therapies. Health Out Res Med.

[14] Barlesi F, Steins M, Horn L, Ready N, Felip E, Borghaei H, Spigel DR, Arrieta O, Antonia S, Fayette J (2016). Long-term outcomes with nivolumab vesrsus docetaxel in patients with advanced NSCLC: checkmate 017 and checkmate 057 2-year update. Asia Pac J Clin Oncol.

[15] Borghaei H, Paz-Ares L, Horn L, Spigel DR, Steins M, Ready NE, Chow LQ, Vokes EE, Felip E, Holgado E (2015). Nivolumab versus docetaxel in advanced nonsquamous non-small-cell lung Cancer. N Engl J Med.

[16] Brahmer J, Reckamp KL, Baas P, Crino L, Eberhardt WE, Poddubskaya E, Antonia S, Pluzanski A, Vokes EE, Holgado E (2015). Nivolumab versus docetaxel in advanced squamous-cell non-small-cell lung Cancer. N Engl J Med.

[17] Fehrenbacher L, Spira A, Ballinger M, Kowanetz M, Vansteenkiste J, Mazieres J, Park K, Smith D, Artal-Cortes A, Lewanski C (2016). Atezolizumab versus docetaxel for patients with previously treated non-small-cell lung cancer (POPLAR): a multicentre, open-label, phase 2 randomised controlled trial. Lancet.

[18] Garassino MC, Martelli O, Broggini M, Farina G, Veronese S, Rulli E, Bianchi F, Bettini A, Longo F, Moscetti L (2013). Erlotinib versus docetaxel as second-line treatment of patients with advanced non-small-cell lung cancer and wild-type EGFR tumours (TAILOR): a randomised controlled trial. Lancet Oncol.

[19] Garon EB, Ciuleanu TE, Arrieta O, Prabhash K, Syrigos KN, Goksel T, Park K, Gorbunova V, Kowalyszyn RD, Pikiel J (2014). Ramucirumab plus docetaxel versus placebo plus docetaxel for second-line treatment of stage IV non-small-cell lung cancer after disease progression on platinum-based therapy (REVEL): a multicentre, double-blind, randomised phase 3 trial. Lancet.

[20] Hanna N, Shepherd FA, Fossella FV, Pereira JR, De Marinis F, von Pawel J, Gatzemeier U, Tsao TC, Pless M, Muller T (2004). Randomized phase III trial of pemetrexed versus docetaxel in patients with non-small-cell lung cancer previously treated with chemotherapy. J Clin Oncol.

[21] Herbst RS, Baas P, Kim DW, Felip E, Perez-Gracia JL, Han JY, Molina J, Kim JH, Arvis CD, Ahn MJ (2016). Pembrolizumab versus docetaxel for previously treated, PD-L1-positive, advanced non-small-cell lung cancer (KEYNOTE-010): a randomised controlled trial. Lancet.

[22] Karampeazis A, Voutsina A, Souglakos J, Kentepozidis N, Giassas S, Christofillakis C, Kotsakis A, Papakotoulas P, Rapti A, Agelidou M (2013). Pemetrexed versus erlotinib in pretreated patients with advanced non-small cell lung cancer: a Hellenic oncology research group (HORG) randomized phase 3 study. Cancer.

[23] Reck M, Kaiser R, Mellemgaard A, Douillard JY, Orlov S, Krzakowski M, von Pawel J, Gottfried M, Bondarenko I, Liao M (2014). Docetaxel plus nintedanib versus docetaxel plus placebo in patients with previously treated non-small-cell lung cancer (LUME-lung 1): a phase 3, double-blind, randomised controlled trial. Lancet Oncol.

[24] Rittmeyer A, Barlesi F, Waterkamp D, Park K, Ciardiello F, von Pawel J, Gadgeel SM, Hida T, Kowalski DM, Dols MC (2017). Atezolizumab versus docetaxel in patients with previously treated non-small-cell lung cancer (OAK): a phase 3, open-label, multicentre randomised controlled trial. Lancet.

[25] Scagliotti G, Hanna N, Fossella F, Sugarman K, Blatter J, Peterson P, Simms L, Shepherd FA (2009). The differential efficacy of pemetrexed according to NSCLC histology: a review of two phase III studies. Oncologist.

[26] Soria JC, Felip E, Cobo M, Lu S, Syrigos K, Lee KH, Goker E, Georgoulias V, Li W, Isla D (2015). Afatinib versus erlotinib as second-line treatment of patients with advanced squamous cell carcinoma of the lung (LUX-lung 8): an open-label randomised controlled phase 3 trial. Lancet Oncol.

[27] Shepherd FA, Rodrigues Pereira J, Ciuleanu T, Tan EH, Hirsh V, Thongprasert S, Campos D, Maoleekoonpiroj S, Smylie M, Martins R (2005). Erlotinib in previously treated non-small-cell lung cancer. N Engl J Med.

[28] Shepherd FA, Dancey J, Ramlau R, Mattson K, Gralla R, O'Rourke M, Levitan N, Gressot L, Vincent M, Burkes R (2000). Prospective randomized trial of docetaxel versus best supportive care in patients with non-small-cell lung cancer previously treated with platinum-based chemotherapy. J Clin Oncol.

[29] Cherny NI, Sullivan R, Dafni U, Kerst JM, Sobrero A, Zielinski C, de Vries EG, Piccart MJ (2015). A standardised, generic, validated approach to stratify the magnitude of clinical benefit that can be anticipated from anti-cancer therapies: the European Society for Medical Oncology magnitude of clinical benefit scale (ESMO-MCBS). Ann Oncol.

[30] Matsuyama R, Reddy S, Smith TJ (2006). Why do patients choose chemotherapy near the end of life? A review of the perspective of those facing death from cancer. J Clin Oncol.

[31] Davis C (2015). Drugs, cancer and end-of-life care: a case study of pharmaceuticalization?. Soc Sci Med (1982).

[32] Beckett P, Callister M, Slade M, Harrison R, Draffan J, Franks K. Sharing information with lung cancer patients: guidance for health care professionals discussing options for patients who have lung cancer. Br Thorac Soc Rep. 2013;5(1):1–27.

[33] Garau M, Shah KK, Mason AR, Wang Q, Towse A, Drummond MF (2011). Using QALYs in cancer: a review of the methodological limitations. Pharmacoeconomics.

[34] Andronis L, Goranitis I, Pirrie S, Pope A, Barton D, Collins S, Daunton A, McLaren D, O'Sullivan JM, Parker C (2017). Cost-effectiveness of zoledronic acid and strontium-89 as bone protecting treatments in addition to chemotherapy in patients with metastatic castrate-refractory prostate cancer: results from the TRAPEZE trial (ISRCTN 12808747). BJU Int.

