# Supplementary material for: Comparative survival benefit of currently licensed second or third line treatments for epidermal growth factor receptor (EGFR) and anaplastic lymphoma kinase (ALK) negative advanced or metastatic non-small cell lung cancer: a systematic review and secondary analysis of trials
Source: BMC Cancer. 2019 Apr 25;19:392. doi: 10.1186/s12885-019-5507-6 (PMC6485098; doi:10.1186/s12885-019-5507-6)
Supplement: Supplementary file 1 — Medline search strategy. (DOCX 13 kb) [file 12885_2019_5507_MOESM1_ESM.docx]

**ADDITIONAL FILE 1:** Medline search strategy

1. (docetaxel or pemetrexed or ramucirumab or erlotinib or nintedanib or afatinib or nivolumab or pembrolizumab or atezolizumab).tw.

2. (non-small cell lung cancer or nsclc).tw.

3. (squamous cell adj4 lung adj4 (cancer or carcinoma)).tw.

4. *Carcinoma, Non-Small-Cell Lung/dt [Drug Therapy]

5. *Carcinoma, Squamous Cell/dt [Drug Therapy]

6. 2 or 3 or 4 or 5

7. 1 and 6

8. (random* or double blind or phase 3 or phase III).tw.

9. randomized controlled trial.pt.

10. 8 or 9

11. 7 and 10

12. limit 11 to english language

13. limit 12 to yr="2000 -Current"

14. (letter or comment or editorial).pt.

15. 13 not 14
