# Supplementary material for: Comparative survival benefit of currently licensed second or third line treatments for epidermal growth factor receptor (EGFR) and anaplastic lymphoma kinase (ALK) negative advanced or metastatic non-small cell lung cancer: a systematic review and secondary analysis of trials
Source: BMC Cancer. 2019 Apr 25;19:392. doi: 10.1186/s12885-019-5507-6 (PMC6485098; doi:10.1186/s12885-019-5507-6)
Supplement: Supplementary file 2 — PRISMA study flow chart (DOCX 28 kb) [file 12885_2019_5507_MOESM2_ESM.docx]

**ADDITIONAL FILE 2:** PRISMA study flow diagram.

**PRISMA flow chart for the selection of studies**

Records identified through database searching (after duplicate removal)
(n = 1948)

Records screened

(n = 1949)

Full-text records assessed for eligibility
(n = 94)

Records included n = 13 (studies, n = 11)

Records excluded at title and abstract level
(n = 1855)

Total number of studies excluded with reasons: (n = 81)

- no relevant KM plot (n=33)

- irrelevant dose regimen (n=8)

- irrelevant population (n=7)

- irrelevant outcomes (n=5)

- irrelevant comparator (n=5)

- irrelevant study design (n=5)

- study protocol only (n=4)

- irrelevant exploratory analyses (n=4)

-insufficient information on EGFR status (n=4)

- duplicate record (n=3)

- irrelevant intervention (n=2)

- Dose escalation study (n=1)

Additional records identified through other sources
(n = 1)

Records identified

(n = 1949)
