# Supplementary material for: Comparative survival benefit of currently licensed second or third line treatments for epidermal growth factor receptor (EGFR) and anaplastic lymphoma kinase (ALK) negative advanced or metastatic non-small cell lung cancer: a systematic review and secondary analysis of trials
Source: BMC Cancer. 2019 Apr 25;19:392. doi: 10.1186/s12885-019-5507-6 (PMC6485098; doi:10.1186/s12885-019-5507-6)
Supplement: Supplementary file 3 — Reconstructed hazard ratios compared to published hazard ratios. (DOCX 19 kb) [file 12885_2019_5507_MOESM3_ESM.docx]

**ADDITIONAL FILE 3:** Comparison of derived and reported hazard ratios.

Studies with mixed histologies, overall survival.

| RECONSTRUCTED | | | REPORTED | | | STUDY |
| --- | --- | --- | --- | --- | --- | --- |
| HR | LCI | UCI | HR | LCI | UCI |  |
| 0.732 | 0.596 | 0.900 | 0.71 | 0.58 | 0.88 | KEYNOTE-010 (2mg dose) |
| 0.715 | 0.529 | 0.965 | 0.73 | 0.53 | 0.99 | POPLAR |
| 0.716 | 0.607 | 0.845 | 0.73 | 0.62 | 0.87 | OAK |
| 0.848 | 0.743 | 0.968 | 0.86 | 0.75 | 0.98 | REVEL |
| 1.266 | 0.949 | 1.689 | 1.28 | 0.95* | 1.96* | TAILOR |
| 0.991 | 0.769 | 1.276 | 0.998 | 0.775 | 1.285 | HORG |
| 0.935 | 0.832 | 1.051 | 0.94 | 0.83 | 1.05 | LUME LUNG-1 |
| 0.981 | 0.807 | 1.192 | 0.99 | 0.82 | 1.2 | Hanna |
| *the values reported were 0.78 95% CI 0.51 – 1.05 for the comparison docetaxel vs erlotinib; the natural logs do not balance and the LCI (0.51) is presumed to be an error. | | | | | | |

Studies with squamous histology, overall survival.

| RECONSTRUCTED | | | REPORTED | | | STUDY |
| --- | --- | --- | --- | --- | --- | --- |
| HR | LCI | UCI | HR | LCI | UCI |  |
| 0.607 | 0.469 | 0.786 | 0.62 | 0.47 | 0.80 | CHECKMATE-017 |
| 0.999 | 0.837 | 1.191 | 1.01 | 0.85 | 1.21 | LUME LUNG -1 |
| 0.885 | 0.694 | 1.130 | 0.883 | 0.692 | 1.127 | REVEL |
| 0.704 | 0.520 | 0.953 | 0.73 | 0.54 | 0.98 | OAK |
| 0.803 | 0.687 | 0.939 | 0.81 | 0.69 | 0.95 | LUXLUNG 8 |
| 1.318 | 0.956 | 1.817 | 1.56 | 1.08 | 2.26 | Hanna |
| *as reported by Scaggliotti et al. | | | | | | |

Studies with non-squamous histology, overall survival.

| RECONSTRUCTED | | | REPORTED | | | STUDY |
| --- | --- | --- | --- | --- | --- | --- |
| HR | LCI | UCI | HR | LCI | UCI |  |
| 0.769 | 0.641 | 0.921 | 0.75 | 0.91 | 0.8 | CHECKMATE-017 |
| 0.828 | 0.699 | 0.982 | 0.83 | 0.7 | 0.99 | LUME LUNG -1 |
| 0.826 | 0.705 | 0.968 | 0.83 | 0.708 | 0.972 | REVEL |
| 0.718 | 0.589 | 0.874 | 0.73 | 0.6 | 0.89 | OAK |
| 1.159 | 0.937 | 1.434 | 1.28 | 1.00 | 1.64 | LUXLUNG 8 |
| 0.769 | 0.641 | 0.921 | 0.75 | 0.91 | 0.8 | Hanna |
| *as reported by Scaggliotti et al. | | | | | | |
