# Supplementary material for: Comparative survival benefit of currently licensed second or third line treatments for epidermal growth factor receptor (EGFR) and anaplastic lymphoma kinase (ALK) negative advanced or metastatic non-small cell lung cancer: a systematic review and secondary analysis of trials
Source: BMC Cancer. 2019 Apr 25;19:392. doi: 10.1186/s12885-019-5507-6 (PMC6485098; doi:10.1186/s12885-019-5507-6)
Supplement: Supplementary file 4 — Results for Progression Free Survival (PFS). (DOCX 4601 kb) [file 12885_2019_5507_MOESM4_ESM.docx]

**ADDITIONAL FILE 4:** Results for progression free survival


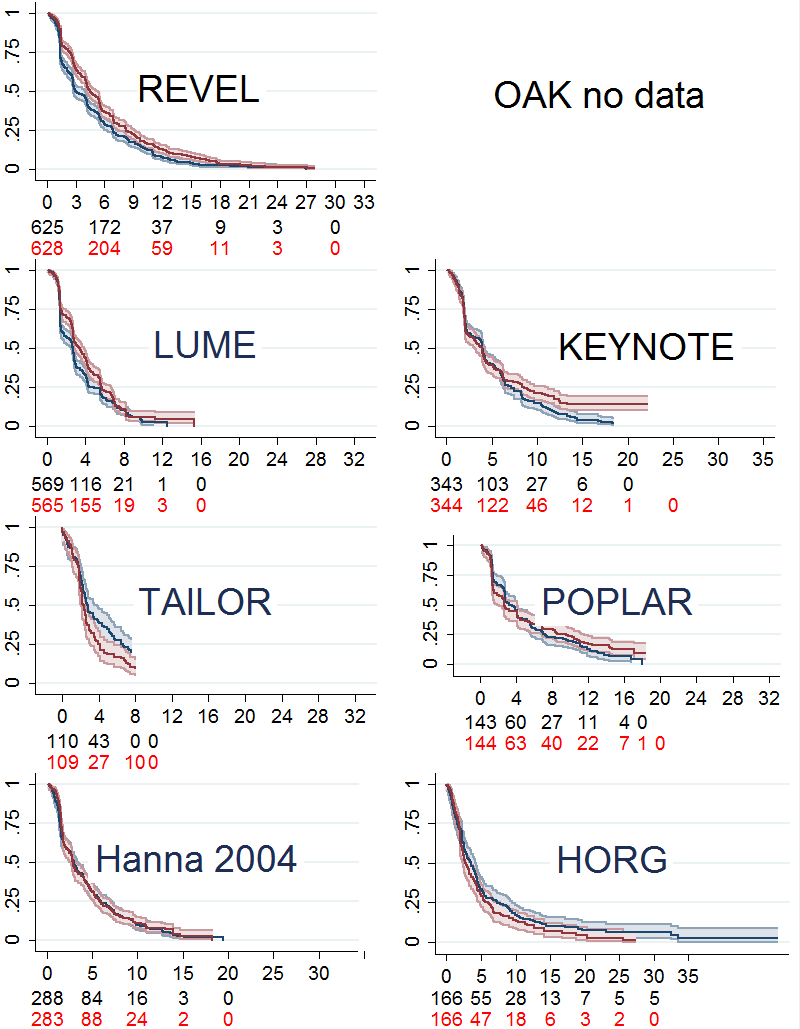


Figure 1 Reconstructed Kaplan-Meier plots (95% CI) of PFS; studies recruiting patients irrespective of tumour histology. *Time axis is months, vertical axis is proportion alive*

**
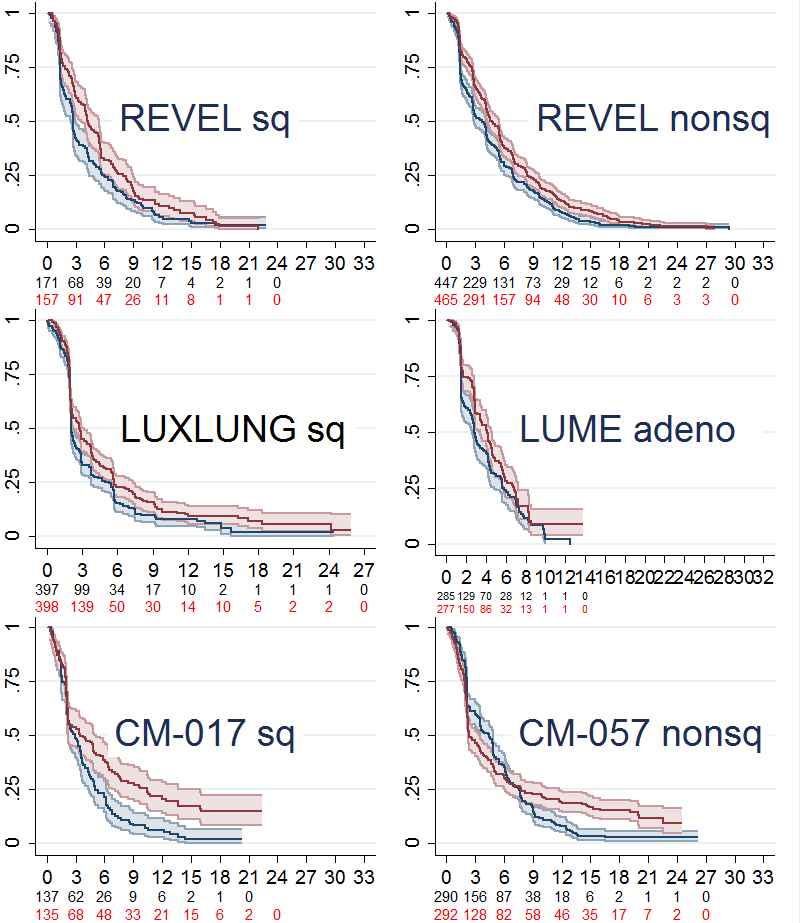
**

Figure 2 Reconstructed Kaplan-Meier plots (95% CI) of PFS; patients stratified by histology. *Time axis is months, vertical axis is proportion alive*

Table 1 Estimates of mean progression free survival based on studies of patients un-stratified by histology

| **TRIAL**  Outcome | **Intervention (n)** | **Control (n)** | **Intervention-control** |
| --- | --- | --- | --- |
| **REVEL** | **Ram + Doc (628)** | **Plac + Doc (625)** |  |
| RMS [95% CI] to 18 mos | 5.92 [5.53 – 6.31] | 4.77 [4.43 – 5.12] | 1.15 [0.63 – 1.60] |
| Modelled Tot Wb | 6.07 | 4.90 | 1.18 |
| **LUME LUNG-1** | **Nin + Doc (565*)** | **Plac + Doc (569*)** |  |
| RMS [95% CI] to 18 mos | *Data to only ~ 15 mos* |  |  |
| Modelled Tot Wb | 4.10 | 3.43 | 0.68 |
| **POPLAR** | **Atezolizumab (144 )** | **Docetaxel (143 )** |  |
| RMS [95% CI] to 18 mos | 5.72 [4.74 – 6.70] | 5.32 [4.45 – 6.19] | 0.40 [-0.91 – 1.71] |
| Modelled Tot Wb | 6.16 | 5.46 | 0.70 |
| **KEYNOTE 01** | **Pembrolizumab (344 )** | **Docetaxel (343)** |  |
| RMS [95% CI] to 18 mos | 6.01 [5.36 – 6.66] | 5.15 [4.63 – 5.68] | 0.86 [0.05 – 1.69] |
| Modelled Tot Wb | 6.11 | 5.21 | 0.90 |
| **TAILOR** | **Erlotinib (109)** | **Docetaxel (110 )** |  |
| RMS [95% CI] to 18 mos | *Data to~ 8 mos only* |  |  |
| Modelled Tot Wb | 3.51 | 4.85 | -1.33 |
| **HORG** | **Erlotinib (166)** | **Pemetrexed (166)** |  |
| RMS [95% CI] to 18 mos | 5.44 [4.62 – 6.27] | 4.76 [3.99 – 5.53] | 0.68 [-0.45 – 1.81] |
| Modelled Tot Wb | 6.37 | 4.99 | 1.38 |
| **Hanna** | **Pemetrexed (283)** | **Docetaxel (288)** |  |
| RMS [95% CI] to 18 mos | 4.44 [3.95 – 4.93] | 4.34 [3.86 – 4.82] | 0.10 [-0.59 – 0.79] |
| Modelled Tot Wb | 4.44 | 4.33 | 0.10 |

Table 2 Estimates of mean progression free survival based patients stratified by squamous histology

| **TRIAL**  Outcome | **Intervention (n)** | **Control (n)** | **Intervention-control** |
| --- | --- | --- | --- |
| **REVEL** | **Ram + Doc (157)** | **Plac + Doc (171)** |  |
| RMS [95% CI] to 20 mos | 5.56 [4.80 – 6.33] | 4.33 [3.67 – 5.00] | 1.23 [0.21 – 2.25] |
| Modelled Tot Wb | 5.57 | 4.40 | 1.17 |
| **LUX-lung 8** | **Afatinib (398)** | **Erlotinib (397)** |  |
| RMS [95% CI] to 20 mos | 5.01 [4.38 – 5.65] | 3.92 [3.39 – 4.45] | 1.09 [0.26 – 1.91] |
| Modelled Tot Wb | 4.78 | 3.73 | 1.06 |
| **LUME LUNG-1** (interim**)** | **Nin + Doc (240)** | **Plac + Doc (247)** |  |
| RMS [95% CI] to 20 mos | *Data to only ~ 12 mos* |  |  |
| Modelled Tot Wb | 3.92 | 3.17 | 0.75 |
| **Checkmate-017** | **Nivolumab (135)** | **Docetaxel (137)** |  |
| RMS [95% CI] to 20 mos | 6.75 [5.55 – 7.95] | 4.12 [3.41 – 4.83] | 2.63 [1.24– 4.02] |
| Modelled Tot Wb | 7.25 | 4.14 | 3.11 |

Table 3 Estimates of mean progression free survival based patients stratified by non-squamous histology

| **TRIAL**  Outcome | **Intervention (n)** | **Control (n)** | **Intervention-control** |
| --- | --- | --- | --- |
| **REVEL** | **Ram (465)** | **Plac + Doc (447)** |  |
| RMS [95% CI] to 14 mos | 5.83 [5.43 – 6.22] | 4.78 [4.40 – 5.15] | 1.05 [0.51 – 1.59] |
| Modelled Tot Wb | 6.26 | 5.02 | 1.25 |
| **LUME LUNG-1** (interim**)** | **Nin + Doc (277)** | **Plac + Doc (285)** |  |
| RMS [95% CI] to 14 mos | 4.67 [4.17 – 5.17] | 3.94 [3.51 – 4.37] | 0.73 [0.08 – 1.39] |
| Modelled Tot Wb | 4.66 | 3.89 | 0.77 |
| **Checkmate_057** | **Nivolumab (292)** | **Docetaxel (290)** |  |
| RMS [95% CI] to 14 mos | 5.25 [4.68 – 5.83] | 5.12 [4.66 – 5.58] | 0.13 [-0.60 – 0.87] |
| Modelled Tot Wb | 6.65 | 5.45 | 1.20 |
