# Supplementary material for: Comparative survival benefit of currently licensed second or third line treatments for epidermal growth factor receptor (EGFR) and anaplastic lymphoma kinase (ALK) negative advanced or metastatic non-small cell lung cancer: a systematic review and secondary analysis of trials
Source: BMC Cancer. 2019 Apr 25;19:392. doi: 10.1186/s12885-019-5507-6 (PMC6485098; doi:10.1186/s12885-019-5507-6)
Supplement: Supplementary file 5 — Restricted mean survival (RMS) results (DOCX 28 kb) [file 12885_2019_5507_MOESM5_ESM.docx]

**ADDITIONAL FILE 5:** RMS estimated over 19 months for un-stratified populations, and over 24 and 27 months for squamous and non-squamous populations respectively.

| 1. Unstratified |
| --- |
| 1. Squamous histology |
| 1. Non-squamous histology |
