# Supplementary material for: Comparative survival benefit of currently licensed second or third line treatments for epidermal growth factor receptor (EGFR) and anaplastic lymphoma kinase (ALK) negative advanced or metastatic non-small cell lung cancer: a systematic review and secondary analysis of trials
Source: BMC Cancer. 2019 Apr 25;19:392. doi: 10.1186/s12885-019-5507-6 (PMC6485098; doi:10.1186/s12885-019-5507-6)
Supplement: Supplementary file 6 — Forest plots of mean difference in RMS and mean difference in total survival (Weibull models). (DOCX 43 kb) [file 12885_2019_5507_MOESM6_ESM.docx]

**ADDITIONAL FILE 6:** Forest plots of mean difference in RMS (A), and mean difference in total survival predicted by Weibull models

1. Forest plot of mean difference in RMD by trial and histology

B Forest plot of mean difference in total survival (Weibull models) by trial and histology
