# Supplementary material for: Comparative survival benefit of currently licensed second or third line treatments for epidermal growth factor receptor (EGFR) and anaplastic lymphoma kinase (ALK) negative advanced or metastatic non-small cell lung cancer: a systematic review and secondary analysis of trials
Source: BMC Cancer. 2019 Apr 25;19:392. doi: 10.1186/s12885-019-5507-6 (PMC6485098; doi:10.1186/s12885-019-5507-6)
Supplement: Supplementary file 7 — Network meta-analysis estimating mean differences in restricted mean survival and overall survival (DOCX 26 kb) [file 12885_2019_5507_MOESM7_ESM.docx]

**ADDITIONAL FILE 7:** Network meta-analysis estimating mean differences in restricted mean survival and overall survival

**Methods:**

Conventional pairwise meta-analysis was done where more than one study reported the same comparison. Mean differences in RMS and in OS were estimated using network meta-analysis (NMA). We examined the distribution of potential effect modifiers across studies so as to assess the transitivity assumption.

Drugs were ranked by the surface under the cumulative ranking curve (SUCRA) method.

Where there were too few studies for each contrast between two treatments, we used a fixed effects model. One network encompassed a single closed loop; here we conducted the side-splitting to test for inconsistency.

The summary estimates (with 95% CIs) for all pair-wise comparisons were developed in league tables in reference to docetaxel. NMA was done using the network suite of commands in Stata^[[1]](#footnote-1)^.

Network diagrams are shown in the results section.

**Results:**

There was an absence of substantial imbalance between studies in potential effect modifiers in networks. The dosages and administration modes of the anchored treatment across trials was consistent.

Months mean differences in OS and RMS between studies are summarised in the league table; comparisons are stratified by SUCRA scores and histology. In all comparisons immunotherapies were ranked higher than all comparator therapies. In analyses pertaining to mixed tumour histology, the network included one closed loop allowing mixed treatment comparison between docetaxel erlotinib and pemetrexed; there was no evidence of inconsistency for the mixed treatment comparisons (doc, erlo, pem) within this loop (p = 0.121 for RMS; p = 0.239 for OS).

**LEAGUE TABLE: Mean survival (months) estimates from network meta-analyses**

| **Mean difference in Weibull predicted overall survival (months) mixed histologies** | | | | | | | | |
| --- | --- | --- | --- | --- | --- | --- | --- | --- |
| Drug | sucra | atezo | pembro | ram | nint | doc | pem | erlot |
| Atezo | 0.92 |  | 0.39 (-4.03,4.81) | 3.55 (0.10,6.99) | 4.56 (1.39,7.72) | 5.43 (2.69,8.16) | 5.73 (2.55,8.91) | 6.74 (2.85,10.64) |
| pembro | 0.89 |  |  | 3.16 (-0.90,7.22) | 4.17 (0.34,8.00) | 5.04 (1.56,8.52) | 5.34 (1.50,9.18) | 6.36 (1.91,10.80) |
| Ram | 0.62 |  |  |  | 1.01 (-1.63,3.65) | 1.88 (-0.22,3.98) | 2.18 (-0.48,4.84) | 3.20 (-0.28,6.67) |
| Nint | 0.47 |  |  |  |  | 0.87 (-0.73,2.47) | 1.17 (-1.11,3.46) | 2.19 (-1.01,5.39) |
| Doc | 0.28 |  |  |  |  |  | 0.30 (-1.33,1.93) | 1.32 (-1.45,4.09) |
| Pem | 0.23 |  |  |  |  |  |  | 1.02 (-1.90,3.94) |
| Erlo | 0.09 |  |  |  |  |  |  |  |
| **Mean difference in restricted mean survival (months) mixed histologies** | | | | | | | | |
| Drug | sucra | atezo | pembro | ram | nint | doc | pem | erlo |
| Atezo | 0.89 |  | 0.00 (-1.40,1.40) | 0.59 (-0.55,1.74) | 1.10 (-0.02,2.22) | 1.58 (0.75,2.41) | 1.81 (0.40,3.22) | 2.21 (0.55,3.86) |
| pembro | 0.87 |  |  | 0.59 (-0.79,1.97) | 1.10 (-0.26,2.46) | 1.58 (0.45,2.71) | 1.80 (0.15,3.46) | 2.20 (0.34,4.07) |
| Ram | 0.67 |  |  |  | 0.51 (-0.58,1.60) | 0.99 (0.20,1.78) | 1.21 (-0.24,2.66) | 1.61 (-0.07,3.29) |
| Nint | 0.48 |  |  |  |  | 0.48 (-0.27,1.23) | 0.70 (-0.72,2.13) | 1.10 (-0.56,2.77) |
| Doc | 0.26 |  |  |  |  |  | 0.22 (-0.99,1.44) | 0.62 (-0.86,2.11) |
| Pem | 0.22 |  |  |  |  |  |  | 0.40 (-0.87,1.67) |
| Erlo | 0.1 |  |  |  |  |  |  |  |
| **Mean difference in Weibull predicted overall survival (months) squamous histology** | | | | | | | | |
| drug | sucra | nivo | atezo | ram | doc | nint | pem |  |
| Nivo | 0.96 |  | 2.43 (-3.35,8.21) | 5.68 (0.72,10.64) | 6.51 (2.50,10.52) | 6.57 (2.08,11.06) | 8.12 (3.57,12.67) |  |
| Atezo | 0.8 |  |  | 3.25 (-1.84,8.34) | 4.08 (-0.09,8.25) | 4.14 (-0.49,8.77) | 5.69 (1.00,10.38) |  |
| Ram | 0.49 |  |  |  | 0.83 (-2.09,3.75) | 0.89 (-2.67,4.45) | 2.44 (-1.19,6.07) |  |
| Doc | 0.36 |  |  |  |  | 0.06 (-1.97,2.09) | 1.61 (-0.54,3.76) |  |
| Nint | 0.33 |  |  |  |  |  | 1.55 (-1.41,4.51) |  |
| Pem | 0.07 |  |  |  |  |  |  |  |
| **Mean difference in restricted mean survival (months) squamous histology** | | | | | | | | |
| Drug | sucra | nivo | atezo | ram | nint | doc |  |  |
| Nivo | 0.95 |  | 1.35 (-1.51,4.20) | 2.65 (0.11,5.18) | 3.10 (0.85,5.35) | 3.61 (1.73,5.49) |  |  |
| Atezo | 0.72 |  |  | 1.30 (-1.45,4.05) | 1.75 (-0.73,4.24) | 2.26 (0.11,4.42) |  |  |
| Ram | 0.43 |  |  |  | 0.45 (-1.65,2.56) | 0.96 (-0.74,2.67) |  |  |
| Nint | 0.31 |  |  |  |  | 0.51 (-0.72,1.74) |  |  |
| Doc | 0.09 |  |  |  |  |  |  |  |
| **Mean difference in Weibull predicted overall survival (months) non-squamous histology** | | | | | | | | |
| Drug | sucra | atezo | nivo | nint | ram | pem | doc |  |
| Atezo | 0.87 |  | 0.96 (-4.27,6.19) | 2.84 (-2.10,7.78) | 3.26 (-1.59,8.11) | 4.33 (-0.37,9.03) | 5.68 (1.61,9.75) |  |
| Nivo | 0.79 |  |  | 1.88 (-2.43,6.19) | 2.30 (-1.90,6.50) | 3.37 (-0.67,7.41) | 4.72 (1.44,8.00) |  |
| Nint | 0.53 |  |  |  | 0.42 (-3.41,4.25) | 1.49 (-2.16,5.14) | 2.84 (0.05,5.63) |  |
| Ram | 0.48 |  |  |  |  | 1.07 (-2.45,4.59) | 2.42 (-0.20,5.04) |  |
| pem | 0.29 |  |  |  |  |  | 1.35 (-1.00,3.70) |  |
| doc | 0.04 |  |  |  |  |  |  |  |

| **Mean difference in restricted mean survival (months) non-squamous histology** | | | | | | | | |
| --- | --- | --- | --- | --- | --- | --- | --- | --- |
| drug | sucra | atezo | nivo | nint | ram | doc |  |  |
| atezo | 0.85 |  | 0.41 (-1.77,2.59) | 1.00 (-1.10,3.10) | 1.16 (-0.84,3.16) | 2.55 (1.00,4.10) |  |  |
| nivo | 0.70 |  |  | 0.59 (-1.51,2.68) | 0.75 (-1.25,2.75) | 2.14 (0.60,3.68) |  |  |
| nint | 0.50 |  |  |  | 0.16 (-1.74,2.07) | 1.55 (0.14,2.97) |  |  |
| ram | 0.44 |  |  |  |  | 1.39 (0.12,2.66) |  |  |
| doc | 0.01 |  |  |  |  |  |  |  |

*atezo = atezolizumab; doc = docetaxel; erlo = erlotinib; nint = nintedanib + docetaxel; nivo = nivolumab; pem = pemetrexed; pembro = pembrolizumab; ram = ramucirumab + docetaxel; sucra =surface under cumulative ranking curve score.*

**Network meta-analysis maps**

| Mixed histology: Mean difference in Weibull predicted overall survival and in RMS. |
| --- |
|  |
| Squamous and non-squamous histology: mean difference in Weibull predicted overall survival |
|  |
| Squamous and non-squamous histology: mean difference in RMS |
|  |

1. Chaimani, A., Higgins, J.P.T., Mavridis, D., Spyridonos, P., Salanti, G.: Graphical tools for network meta-analysis in STATA. PLoS ONE. 8, e76654 (2013). doi:10.1371/journal.pone.0076654 [↑](#footnote-ref-1)
