# Supplementary material for: Comparative survival benefit of currently licensed second or third line treatments for epidermal growth factor receptor (EGFR) and anaplastic lymphoma kinase (ALK) negative advanced or metastatic non-small cell lung cancer: a systematic review and secondary analysis of trials
Source: BMC Cancer. 2019 Apr 25;19:392. doi: 10.1186/s12885-019-5507-6 (PMC6485098; doi:10.1186/s12885-019-5507-6)
Supplement: Supplementary file 8 — Association of survival estimates and reported medians and hazard ratios. (DOCX 32 kb) [file 12885_2019_5507_MOESM8_ESM.docx]

**ADDITIONAL FILE 8:** Association of survival estimates and reported medians and hazard ratios.

|  |  |
| --- | --- |
|  |  |
|  |  |

HRs from TAILOR and the post hoc analysis by Scagliotti et al. were also omitted as errors / outliers.
